# Consumers’ Perspectives on Alternative Short Food Supply Chains Based on Social Media: A Focus Group Study in Spain

**DOI:** 10.3390/foods9010022

**Published:** 2019-12-24

**Authors:** Ahmed Elghannam, Francisco J. Mesias, Miguel Escribano, Lina Fouad, Andres Horrillo, Alfredo J. Escribano

**Affiliations:** 1 Faculty of Agriculture, University of Extremadura, 06006 Extremadura, Spain; ahorrilly@gmail.com; 2 Faculty of Agriculture, Damanhour University, 22511 Damanhour, Egypt; leenafouad.lf@gmail.com; 3 Faculty of Veterinary Medicine, University of Extremadura, 10003 Extremadura, Spain; mescriba@unex.es; 4 Independent Researcher & Consultant, 10005 Cáceres, Spain; ajescc@gmail.com

**Keywords:** short food supply chains, focus groups, social media, alternative supply chains, electronic word of mouth (eWOM), social media marketing

## Abstract

Nowadays, an increasing number of consumers are demanding more information and more direct contact with food producers in order to avoid the various intermediaries in the supply chain, thus improving food traceability and price transfer. This has led to the development of more direct (short) food supply chains (SFSCs). Although consumer preferences to use SFSCs rather than traditional (long) supply chains have been widely researched in the literature, this study brings a new approach with the use of social media sites to build online SFSCs. A focus group approach with a total of 32 participants was used in this study with the main objective to understand consumers’ awareness and acceptance of SFSCs. Special attention was given to the use of social media and electronic word of mouth (eWOM) as new approaches to support the creation of such alternative channels.

## 1. Introduction

The agrifood sector, which includes not only the primary sector, agro-industry and distribution, but also all members of society as consumers [1], plays an enormous socio-economic [2] and environmental role in Spain and across Europe, as it is the only employment alternative in many rural areas. It contributes to reducing depopulation and maintaining high nature value ecosystems—those of relevance from an environmental point of view [3]—that would otherwise be in danger of disappearing.

In recent years, this sector has undergone several changes, one of the most important of which is related to the concentration of some of the players in the supply chain [4]. This means that large distribution companies have increasingly become the main link between producers and consumers [5]. This situation creates an imbalance in the producer–distributor relationship, as producers see their bargaining power reduced with respect to the terms of sale of their products. In this new context, distributors are in control of the process, also having developed the capability to influence and conduct demand [6].

This generates dissatisfaction for both producers and consumers. The former are forced to significantly lower the prices for their products and find neither a stable market nor a return on their activity [5], which constrains their investment capacity and undermines their ability to maintain activity. The latter perceive that the reduced prices charged by producers at the farm level are not reflected in lower prices for the food products they purchase [7].

Apart from economic dissatisfaction, conventional agrifood supply chains have often been criticised for their adverse environmental and social effects [8]. Globalization and the development of international logistic networks increasingly facilitate the marketing of food from different countries and thus outside their traditional selling periods. This can be seen as positive by many consumers, who find more variety in food at more affordable prices. However, it also entails negative aspects due to the environmental impact of transport, as well as the loss of income of local producers who are not able to compete in these scenarios.

This has led to an increasing number of consumers demanding more information regarding the origin, safety and wholesomeness of the food they purchase [9]. They currently seek more direct contact with food producers, thus avoiding the various intermediaries in the supply chain between their food and them, and facilitating food traceability and better price transfer between producers and consumers. This situation has led to the development of new types of more direct chains between producers and consumers, which have arisen as a promising sustainable alternative in terms of economic, social and environmental benefits [10] and are increasingly gaining ground across Europe.

These new marketing channels are called short food supply chains (SFSC) and their development can generate great opportunities for agricultural and agro-industrial producers, as SFSCs would put producers and consumers in direct contact, improving marketing and increasing added value. They could also contribute to improved food traceability, while at the same time creating conditions for a better transfer of the prices between producers and consumers [11]. Nevertheless, in certain conditions, the use of SFSCs could increase the final price paid by consumers, especially if logistic chains are not optimised or if SFSCs are over-extended.

The literature shows various definitions of SFSCs [5,7,12], with one of the most accepted ones being that of the Council of Europe, which defines SFSCs as “channels made up of a limited number of economic agents, committed to cooperation, local economic development and socio-economic relations between producers and consumers in a nearby geographical area” [12]. This definition can be seen to confine SFSCs to local production chains; however, the concept of a short channel can be extended spatially [13], and therefore, broader definitions that can explain the full concept are needed. In this context [5], we have defined SFSCs as chains in which the number of intermediaries is equal to or fewer than one, whether the transaction takes place through online or offline outlets. This definition could cover various types of on-farm and off-farm SFSCs. According to [12], on-farm schemes refer to those where consumers go themselves to the production site to purchase the products directly from the farmer, which would include farm shops, farm-based hospitality schemes, roadside sale sites, pick-your-own schemes, etc. Farmers can also use off-farm schemes to sell their products to local consumers in nearby neighbourhoods, as in the case of farmers’ markets, shops owned by farmers, food festivals and fairs, and farm-based delivery schemes. SFSCs can also deal with non-local sales, in particular direct internet sales/long distance farm-based delivery schemes [12].

SFSCs have been spreading all over the world as they have often been seen as capable of making a valuable contribution to the provisioning of positive externalities from the environmental, economic, and social points of view [14], which can shape consumer’s views towards SFSCs. In fact, the European Common Agricultural Policy 2014–2020 has adopted the promotion of SFSCs and local food within the II Pillar to provide a publicly funded stimulus for sustainable development. However, such externalities depend on many factors that make the abovementioned benefits differ from one chain to another [14].

Other studies [7] distinguish two main types of short chains (on-line and off-line) according to the use of the Internet. The short on-line channels allow one to buy products directly through the web or simply to obtain help or to offer information in real time about the products on offer. In these online models, proximity does not matter as online-buyers can purchase products even from outside their country. On the other hand, short off-line channels are channels that do not have an Internet sales service. In the current context, with an increasingly widespread use of the Internet, off-line channels are losing relevance compared to online channels, where such concepts as direct sales through the Internet with home delivery or consumers’ groups with on-line contact and feedback between producer and consumer are booming.

Within this context, the development of social media, with its capability to build networks of users [1,15,16,17,18,19,20,21,22], opens up new opportunities to create SFSCs. Social media can allow producers to better identify their customers’ socio-demographics, therefore improving the definition of their target segments and the adaptation of their marketing strategies to meet their demands [11]. Nevertheless, the perception of some consumers towards social media, as social but non-commercial platforms, can hinder their use [5,23].

Even though consumer preferences towards the use of SFSCs vs. traditional food chains have been widely researched in the literature [24,25], this study introduces a new approach through the use of social media sites to build online SFSCs. The aim of this paper is to analyse consumer knowledge and acceptance of short food supply chains, but focusing on the use of social media and online word of mouth, as new tools to foster the development of SFSCs. A qualitative methodology was adopted for the purposes of this piece of research, as it has been frequently used in previous studies dealing with consumer perceptions on SFSCs in different countries [11,26].

## 2. Materials and Methods

### 2.1. Focus Group

Due to the originality of the topic under study, where most consumers would not have had direct experience, it was decided to use a qualitative approach based on focus groups. The research team considered that in this way participants would be able to provide their subconscious motivations and beliefs about this issue, as these subjective aspects could not appear through direct questioning [27].

Among qualitative techniques, focus groups have been widely used in market research dealing with a range of consumer-related marketing topics and facilitating an in-depth understanding of certain issues [28]. In this sense, focus groups are considered one of the most important methodologies generally used in marketing and consumer research [29]. The main advantage of the focus groups is that they allow much more freedom of speech among participants, encouraging them to interact, debate and exchange views during the discussion [30].

Generally, this approach is useful because it provides a realistic and practical vision, breaking up with traditional vertical and unidirectional patterns of knowledge transfer with the purpose of achieving a multidirectional flow of information between research institutions, productive sectors and consumers. Numerous studies can be found on subjects as diverse as the perception of food packaging [31], the development of new food brands for agroforestry systems, or the study of the effect of information on preferences [32].

### 2.2. Participants

For the purposes of this study, focus group sessions were carried out in various parts of the Extremadura region (SW Spain). A convenience sample of 32 participants was recruited and then divided into four different groups (see Table 1). They were selected as regular shoppers in their households and frequent users of at least one social media platform. This number of participants is in line with other studies in the field [33,34]. The four sessions were conducted during June 2018, and each of them lasted 35 min on average. At the beginning of each session, the moderator explained the objectives of the study to the participants, showed how the activity would be carried out and how the data would be processed. Participants were required to provide honest and straightforward answers about their thoughts, as the study was about their perceptions and there could be no right or wrong answers [33,34].

### 2.3. Focus Group Structure

**Figure 1:**
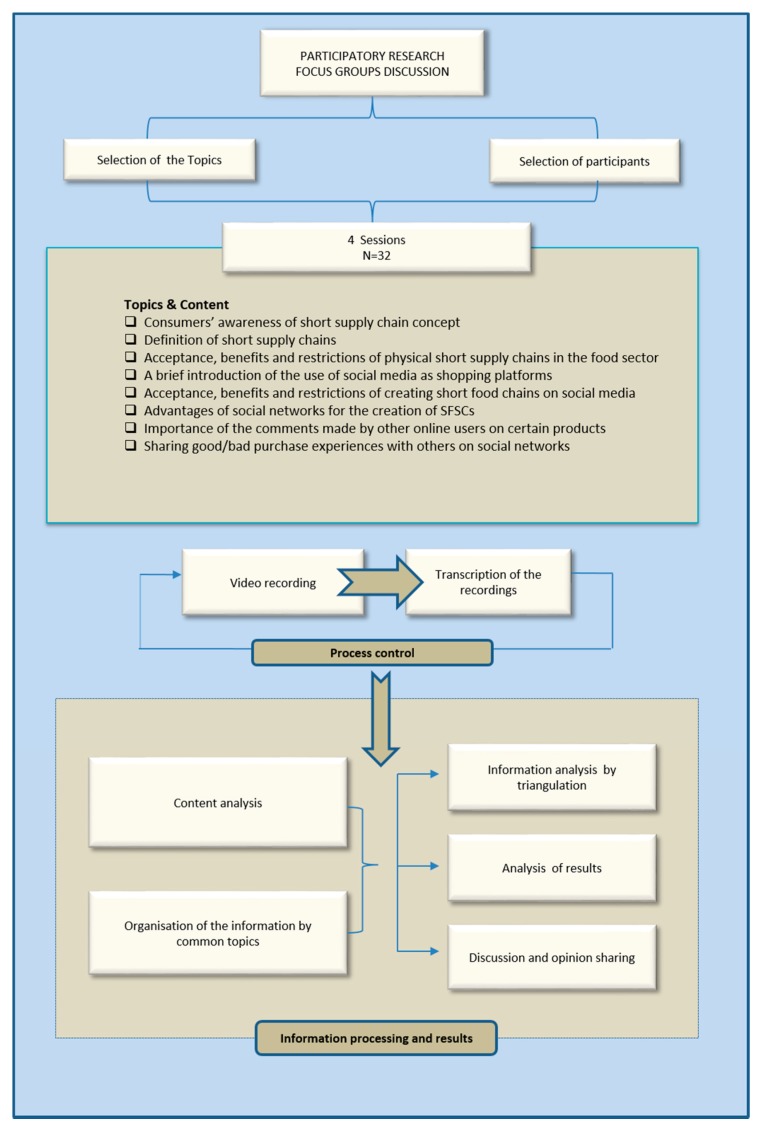
Structure of the focus group and the methodological approach.

The focus group development and structure can be seen in Figure 1. The guideline was divided into various sections:

Section 1: this section related to consumer awareness of the short supply chain concept, as they were asked to use their own words to define the concept of short supply chain.

Section 2: after respondents gave their answers on the first section, the second section was devoted to providing a general and simple definition of short supply chains for those who were not aware of the precise meaning of the concept.

Section 3: within this section, participants were asked to provide their own opinion (acceptance, benefits and restrictions) on the idea of creating short supply chains within the agrifood sector.

Section 4: provided a brief introduction about the use of social media as shopping platforms. Participants were informed that “nowadays, social media like Instagram, Facebook and Twitter are developed and not only serve as advertising channels, but also offer the necessary tools to operate as sales platforms. In this sense, consumers can buy directly through social platforms, which is known as social media shopping. The entire process from product selection to delivery can be done and tracked on the social media networks. Many companies have already launched this type of sales strategy, including some food companies. Therefore, small producers can take advantage of social media to build short food supply chains…” In addition, some actual examples of “shop now buttons” taken from Instagram and Facebook were shown to them in order to show them the bigger picture.

Section 5: was related to the level of acceptance, the perceived benefits and limitations of the participants, as they were asked to mention their points of view on whether the use of social media as a platform for the creation of short food supply chains was positive or negative.

Section 6: focused on the advantages of social media for small producers to create short food supply chains.

Finally, Sections 7 and 8 were associated with the importance of online users’ comments and asked whether the participants, as users of social media platforms, tended to share their own experiences of purchasing with others.

### 2.4. Data Analysis

Focus groups were conducted in Spanish to ensure that all participants were confident to express themselves in their mother tongue. All focus group discussions were recorded and transcribed. The transcripts were then translated into English and imported into ATLAS.Ti v.8 for content analysis. Data analysis was carried out using analyst triangulation, a methodology frequently used in qualitative research in order to improve the robustness of the results.

During the first stage, the data were arranged into common topics. Subsequently, the concepts that were frequently quoted during the focus groups were classified into blocks. Finally, the frequency of mentions for each block was calculated in order to show its relative importance, as it is generally assumed that the item that receives the highest frequency of comments is considered more relevant than the others. To do so, and instead of just dividing the total number of mentions for each block by the total number of participants—which may be unreliable due to the fact that some participants contributed with more than one comment—we divided the frequency of mentions of a block by the total number of mentions of all blocks related to a certain topic.

## 3. Results and Discussion

### 3.1. Consumer Awareness of the Short Supply Chain Concept

The majority of the participants seemed to be unfamiliar with the concept of short supply chain, as no more than 7% of them could provide a general definition of the concept. The definition provided by those who were familiarised was simply that it means “purchasing directly from the farmer”, while the rest of the participants were totally unaware. At that point, the moderator provided a clear definition in order to put consumers into the picture and to begin the debate.

Once participants were informed about the concept, they began to react to this type of alternative channel: “great idea, because that way you know what you are buying”, “you buy a little bit cheaper and the profit goes to the producer in the first place” and “I think it would gain its own market share, especially in the case of local products with high added value”. Interestingly, the situation reversed in comparison with their level of knowledge, as only less than 7% of them stated that they were reluctant to adopt this initiative to purchase their products.

Even though there is much evidence of the growing interest in the development of short food supply chains in different western countries [7,24,25], the rate of growth of these structures in the Spanish market remains below that of other neighbouring regions [7]. Specially in the most rural regions—such as Extremadura, where the study took place—many consumers are still very close to their homelands, and in many cases have agricultural roots. This may lead to the lack of need and interest in SFSCs, which is reflected in this study.

However, the main attributes concerning short channels (local products, reduced number of intermediaries, competitive prices for consumers…) [5,35,36] were mentioned in the discussions, which may indicate that although not many consumers are familiar with the term, they are aware of the underlying concepts.

It is also noteworthy to highlight that although consumers addressed the fact that they would trust short supply chains for their purchases, some other concerns/mentions cropped up during the sessions such as: “Should they need to ensure a lesser use of transports?”, “What if I buy a product online from a producer in another country?” and “Will these producers be paying the applicable taxes to the government?”.

### 3.2. Consumer Reflections on the Use of Physical and Social Media-Based Short Supply Chains in the Food Industry

The results showed some controversial opinions, when it came to buying food in physical or social media-based short chains, as the participants provided a lot of positive and negative comments relating to both models. Table 2 represents consumer comments on the use of short supply chains for their food purchases. The findings revealed that more than 64% of the overall comments on those alternative food channels were positive. Respondents think that food is the product with most marketing potential through short supply chains. In this sense, a total of 21.4% of the comments stated that they would accept buying from SFSCs, based on the fact that products do not go through a lot of middlemen (14.3%) and that SFSCs inspire confidence (7.1%).

In contrast, the main negative comments were the perception that SFSCs would lead to increased prices in some cases (14.3%), the increase of the efforts required from the consumer as the purchase cannot be made all at once (7.1%). However, although food products coming from short supply chains sometimes have a higher price, this usually happens when such products are dressed with a storytelling atmosphere surrounding them (e.g., in local/organic markets). However, these products can be found at accessible prices (even lower ones), as it happens in rural areas (e.g., in weekly markets).

One of the points of discussion was the meanings that the various concepts and terms associated with the SFSCs elicited in the minds of consumers. Some of them were contradictory meanings and their effects on consumers were different, if presented alone or in combination. The results also showed that SFSCs are particularly associated with “local”. In many cases, SFSCs are considered to be “sustainable”, in terms of having better environmental performance and raising awareness of local farmers and farming [25,37], as well as being widely promoted in agricultural policy, particularly in the European Union [10]. Consumers support SFSCs as an option of sustainable consumption, as it means local for many of them, which in turn elicits positive effects on economic, social and environmental aspects [38,39].

However, the empirical evidence of the impact of the various categories of marketing channels (including SFSCs) on the social and environmental aspects of sustainability is very limited [40]. Thus, consumer preferences and purchasing decisions are based more on concepts and beliefs than on the effects of those marketing channels. SFSCs would be insufficient for informed and alternative consumers concerned about food miles (the distance that foodstuff travels between the production location and the consumption marketplace [41]), as it happens with other “sustainability” or green attributes. In other types of “alternative” foods, e.g., organic, consumers prefer to buy organic food from smaller, alternative locations, despite their higher prices [42]. From an alternative consumer’s viewpoint, the fact that SFSCs belonged to the mainstream/conventional food system would be perceived as a conventionalisation of the SFSC scheme.

For sustainability-concerned consumers, the key is not the category of supply chain but the effects of the supply chain on the sustainability of the products. In this sense, transportation accounted for 17.43% of the total energy consumed by the Spanish food sector in 2000 [43]. Despite the belief that SFSC products cause lower environmental impacts, foods marketed under this scheme sometimes prove to be inefficient due to the small number of products that are distributed per vehicle or unit of energy. In this regard, [43] found that most of the abovementioned 17.43% of energy consumed by transport comes from road transport due to their lower energy efficiency per load transported. On the contrary, the concentration of supply can lead to lower emissions of GHGs (green house gases). Such concentration does not depend on the SFSC scheme, either short or “large”. In fact, [40] found that “longer” supply channels generate lower environmental impacts per unit of production when measured in terms of carbon footprint and food miles.

From a consumer perspective, these contradicting attributes may be too hard to understand, and therefore labelling strategies play a relevant role as the combination of the various attributes (SFSCs with food miles and others) can drive purchasing decisions [44].

Table 3 shows the comments made regarding the use of social media-based SFSCs. As opposed to the physical model, the percentage of positive comments was lower than that of the negative comments. In particular, the results revealed that social media-based SFSCs can benefit from their alignment with young people (5%). However, most of the attendants indicated they would accept those chains only if provided certain conditions were met, for example, if quality was guaranteed (10%) or if they were familiar with the producer (15%). Whereas the negative comments mainly emphasised the trust issue (30%) and unacceptability (10%) of online food purchases, 10% of the total mentions referred to the use of social media as a source of information and not a purchasing channel.

Trust is one of the main issues in food consumption and purchase, due to the different scandals that have been spread in recent years [45] and that remain in the memory of consumers for a long time (e.g., beef after BSE by [46]). In this sense, building trust must be a top priority for producers selling through social media platforms. Our findings showed that caution must be exercised in terms of trust and professionalism (30% of the comments highlighted this aspect). Selling through online platforms could increase the psychological distance between producer and consumer, especially in those consumers who are not very used to purchasing online (those before the Millennials and the Z-generation), which could weaken consumer perception of short supply chains, especially regarding its “local” and environmentally-friendly side (they are produced farther away and require more transport) [37]. For all of the above reasons, the building of trust in an online environment (e-trust) is a key factor for the consolidation of these marketing channels [1,5,47].

It can be said that logistics play a key role in the perception of SFSCs, as many consumers are now used to just-in-time and fast delivery services, especially those consumers who are/will be more likely to purchase foods through social media. Moreover, logistics is also a significant issue for many producers, as they are not used to efficiently managing this supply chain link. Additionally, the delivery service significantly shapes the image of a company (producer) as perceived by the consumer.

### 3.3. Advantages of Social Media as a Driver for the Creation of Short Food Chains

**Figure 2:**
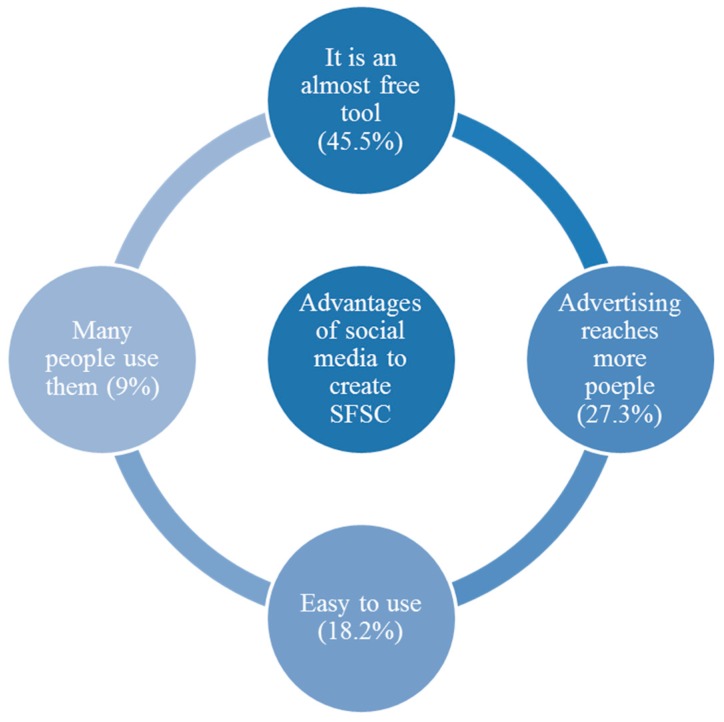
Advantages that enable social media to create SFSCs.

When participants were asked to mention the strengths that would convert social media into a successful short food supply chain (Figure 2), the most mentioned issue was its cost-free nature. Attendants also indicated that free-of-charge service could be the first driver for small producers to create their online short food chains on social media. They provided comments like: “It is almost free, you just take a photo and post it; less expenditure on infrastructure; free advertising”, in addition to the possibility of reaching a higher number of potential customers (27.3%), where their comments were “if you like a post, it goes out to thousands of people; advertising reaches more people”, and followed to a lesser extent by the ease of use for non-expert users.

In fact, back in 2013, the Spanish Ministry of Food and Agriculture started to advise SMEs (small and medium-sized enterprises) that they should develop marketing strategies through the use of websites and social media, which were considered to be affordable tools that were easy-to-use by non-experts [7]. As stated by [5], social media could be used by producers to create new short channels where they could advertise and sell their products at low-cost and in a direct way. Social media is also a widespread tool, with users accounting for more than 50% of the population, even in developing countries [11], which makes their potential reach much greater.

From the point of view of the producers, social media can also improve their marketing profile, for example by helping them build brand image through the investment in online brand positioning strategies. In this context, social media could be the way for producers to create emotional and informational links with their customers. Once consumers perceive that this relationship satisfies their needs, it would be natural for them to commit to this website, thus becoming co-creators of a strong brand for the company [15]. The possibility of having direct contact with customers is also interesting, as it would have implications for customer service, ideas about new product developments and feedback on the aspects needing improvement. Moreover, this last point would allow one to work on the previously mentioned trust.

### 3.4. Electronic Word of Mouth (e-WOM)

**Figure 3:**
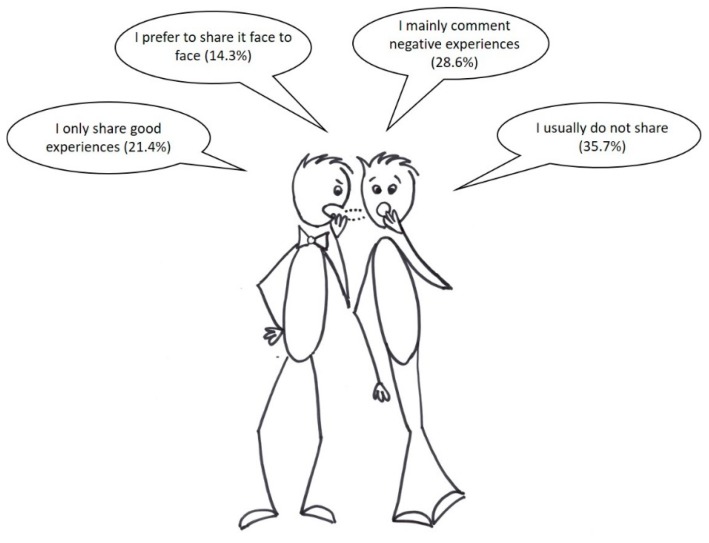
Sharing previous purchasing experiences on social media.

The analysis of our data revealed that for 100% of the participants, the feedback/reviews/assessments made by other social media users on certain products are crucial when it comes to making a purchase decision: “we go through other people’s comments; word of mouth is very important for me”. However, in Figure 3 we can see that a high percentage (35.7%) of the participants do not share any feedback on the products they buy, whereas 50% of them tend to share their experiences with previous purchases with other users on social media. In particular, they seem to share the negative comments more than the positive ones.

Various authors have stated that e-WOM is one of the most relevant attributes of social marketing [23,48]. Social media opens up the possibility for consumers to share their purchasing and consumption experiences, but also to take into account the assessments and recommendations of other Internet users. This is an opportunity for many food producers to build their business reputation and secure customer trust [19], as, up until now, they have not been able to access their customers and their feedback, which perhaps are not very numerous and are probably geographically dispersed. However, it may also bring a risk for companies that do not properly manage their relationship with their customers, as negative motivations are more likely to generate user response than positive motivations.

E-WOM is also associated with trust, as customers who trust social media’s commercial sites are more likely to post positive word-of-mouth comments and purchase from these sites [48]. In this context, the existence of word-of-mouth referrals can also increase customer’s trust and therefore, purchasing intentions [17]. It has also been found that consumers are willing to spend time reading reviews, not only for the sake of information, but also because in this way they get to understand the experience of other users, therefore providing a kind of social experience [49]. Businesses should be aware of this use of social media sites in order to persuade consumers to share information while, at the same time, generating interactions with other users.

## 4. Conclusions

The current study was an attempt to shed light on the degree to which consumers were aware of social media-based SFSCs and their attitudes towards them. Our research has found that the main opportunities for social media to succeed as SFSCs are their low cost, their potential to be adopted by young consumers and their ability to collect key information about customers (marketing-advertising campaigns and product development) and receive real-time feedback. It has also been found that these short food chains can offer a wide range of food products, thus adapting to the new habits of that part of society that is more interested in being informed about the origin of the products they buy. In this sense, consumers must receive enough information about the products and the company, as well as guaranteed certifications so that they can make reliable purchasing decisions.

This study also highlighted the relevance of eWOM in consumer perception, as all attendees had received feedback from others who had previously purchased the same product. However, it was also noted that only one in five of them shared positive purchasing experiences on social media. This leads us to recommend that producers encourage their customers to assess the products they buy online in return for special promotions or future discounts.

Mistrust and the inconvenience of the purchasing process through social media-based short food chains were the main constraints for participants. In addition, the authors would like to add logistics excellence, which is deemed to be key for very demanding consumers—especially those more likely to purchase through social media. However, many farmers are unfamiliar with this supply chain link. In spite of this, social media exposure is crucial for small producers, as it can help them approach potential customers who tend to use social media as a non-purchase information source and may thus become future customers.

This piece of research, despite its exploratory nature, could help open up new opportunities for further quantitative and qualitative studies at both national and cross-cultural levels. One piece of research could focus on exploring small producers and their awareness and willingness to use social media as a tool with potential to create short food chains. We would also suggest performing more insight research using quantitative research techniques in order to address the role of electronic word of mouth in consumer intention to purchase food through social media-based short food supply chains.

## Figures and Tables

**Table 1:** Socio-demographic characteristics of participants.

Characteristics		Percentage
Sex	Male	25
	Female	75
Age	18:30	28
	31:50	31
	>50	40
Academic level	Primary	22
	University	78
Occupation	Student	12.5
	Unemployed	31.3
	Employed	56.2

**Table 2:** Consumers’ comments on the use of physical short supply chains.

Blocks	Main Quotes	% of Total Mentions
Acceptance to buy from SFSCs	Well, you buy food that offers a guarantee; I would buy fresh food like meat, chicken, vegetables and fruit; I would use this initiative in the case of food; the more of these initiatives, the better	21.4
Appropriateness for food products	I think they can get their own market share in the food sector; It would work properly with seasonal and local food products with added value e.g., organic; we already buy organic products from the producer through a consumer group.	21.4
Product may become more expensive	If the short channel is very limited, the product becomes more expensive; if the product is a piece of delicatessen, the short chain becomes expensive	14.3
It must be local	It has to be much more local; those chains go against globalisation	14.3
Less damage to the product	The product suffers less damage; the product is in much better condition than products that have gone through a lot of middlemen	14.3
Trust perception	Perceived trust and guarantee	7.1
You can’t buy everything at once	If you want to make the purchase worth one month, you need to go to 20 thousand places.	7.1

**Table 3:** Consumers’ comments on the use of social media-based SFSCs.

Blocks	Main Quotes	% of Total Mentions
Trust	It doesn’t convey much confidence; trust has to be gained little by little; it is an unprofessional channel; with the creation of trust any product can be bought even if it is sold online; the word that has come out a thousand times in that conversation is trust; they can get to transmit the trust	30
Renowned producer/brand	I would accept the idea if it were from an acquaintance or a good brand; on the internet I don’t know what I’m buying or who I’m buying from, unless it’s a product that I can only buy online; I have no problem buying a food online from a producer I know	15
Not a good idea	I would not buy at the moment; may be in the future. It is not worth it; I’m not going to do it	15
Non-purchase information source	I would not buy on social media, however, if the link takes me to a website, that is something else; I would Google them later on and look for the company	10
Great idea	This is a great idea; a good idea	10
Quality guarantee	I trust the PDO; they have to provide some quality assurance	10
I like to purchase in person	I like to do my shopping in a supermarket; I trust buying in supermarkets	10
For younger consumers	They can reach a younger segment of the population	5
